# FAM107A Inactivation Associated with Promoter Methylation Affects Prostate Cancer Progression through the FAK/PI3K/AKT Pathway

**DOI:** 10.3390/cancers14163915

**Published:** 2022-08-13

**Authors:** Shuai Ke, Zelin Liu, Qinghua Wang, Guanzhong Zhai, Haoren Shao, Xi Yu, Jia Guo

**Affiliations:** Department of Urology, Renmin Hospital of Wuhan University, 99 Zhangzhidong Road, Wuhan 430060, China

**Keywords:** FAM107A, prostate cancer, methylation, EMT, FAK/PI3K/AKT pathway

## Abstract

**Simple Summary:**

Prostate cancer (PCa) is a common male malignancy. FAM107A, or actin-associated protein, is commonly downregulated in PCa and is associated with a poor patient prognosis. We investigated the role of FAM107A in PCa and found that downregulation of FAM107A expression was caused by hypermethylation of CpG islands, and DNA methyltransferase 1 (DNMT1) was involved in maintaining hypermethylation. Mechanistically, FAM107A regulated PCa cell growth through the FAK/PI3K/AKT signaling pathway. Therefore, FAM107A overexpression may represent a potential treatment for PCa, while therapies targeting epigenetic events that regulate FAM107A expression may also be an effective strategy for PCa treatment.

**Abstract:**

Prostate cancer (PCa) is one of the most common cancers and is the second leading cause of mortality in men. Studies exploring novel therapeutic methods are urgently needed. FAM107A, a coding gene located in the short arm of chromosome3, is generally downregulated in PCa and is associated with a poor prognosis. However, the downregulation of FAM107A in PCa and the mechanism of its action remain challenging to determine. This investigation found that downregulation of FAM107A expression in PCa was caused by hypermethylation of CpG islands. Furthermore, DNA methyltransferase 1 (DNMT1) was involved in maintaining hypermethylation. Mechanistically, overexpression of FAM107A inhibits tumor cell proliferation, migration, invasion and promotes apoptosis through the FAK/PI3K/AKT signaling pathway, indicating that FAM107A may be a molecular brake of FAK/PI3K/AKT signaling, thus limiting the active state of the FAK/PI3K/AKT pathway. These findings will contribute to a better understanding of the effect of FAM107A in PCa, and FAM107A may represent a new therapeutic target for PCa.

## 1. Introduction

PCa is a disease characterized by aggressive growth and poor prognosis and has become the most common malignancy among men in developed countries. Currently, PCa is the second-largest cause of cancer-related death in the United States [1]. The incidence of PCa in China is growing faster than that of other cancers, and the factors for its growth rate are not apparent [2]. Surgery or androgen deprivation therapy has shown some benefit in the treatment of early PCa [3]. However, some patients inevitably progress to the castration-resistant stage of PCa, the main fatal stage of PCa. Thus, the identification of new therapeutic targets is critical for treating patients with advanced PCa.

Multiple tumor suppressor genes (TSGs) are present on the short arm (3p) of human chromosome 3, and inactivation or loss of TSGs is frequently linked to tumor development. Common mechanisms of TSG inactivation include transcriptional regulation and posttranslational modification and epigenetic silencing resulting from promoter hypermethylation [4,5]. FAM107A (actin-associated protein), also known as DRR1 (downregulated in renal cell carcinoma) and TU3A (Tohoku University cDNA clone A on chromosome 3), is located in the 3p region [6,7].

FAM107A is a protein-coding gene that encodes a protein of 144 amino acids. Expression of this protein can be widely detected in normal human tissues and plays an essential role in the development of the human nervous system, promoting the motility of neural progenitor cells [8]. It also has the potential to be a target for the development of metabolic disorders of obesity due to its role in the regulation of lipid metabolism [9]. Moreover, abnormal expression of FAM107A is also involved in the progression of multiple tumors. Specifically, overexpressed FAM107A reduces the invasiveness of renal cancer cells and inhibits their growth ability in immunodeficient mice [10]. Furthermore, FAM107A overexpression has been shown to act as an inhibitor of tumor growth and invasion in bladder cancer [11]. It can also inhibit the transformation of the G1/S phase of neuroblastoma and colorectal cancer cells [12]. The level of FAM107A protein in laryngeal squamous cell carcinoma is lower than that in normal tissues due to hypermethylation of the promoter region [13]. In general, FAM107A is closely associated with cancer development, but how FAM107A affects PCa remains unclear. The relationship between FAM107A and PCa deserves further investigation.

Here, we provide clear evidence that FAM107A acts as a downregulated gene in PCa. Furthermore, promoter hypermethylation is the epigenetic mechanism that leads to its downregulation in PCa. Overexpression of FAM107A inhibits the FAK/PI3K/AKT pathway, prevents PCa cell proliferation, and promotes apoptosis. This research can hopefully bridge the gap between the current function and the regulation of FAM107A in PCa, while providing a rationale for further exploration of therapeutic targets in PCa.

## 2. Materials and Methods

### 2.1. Clinical Specimens and Cell Culture

Both PCa tissues and cancer adjacent tissues used in this experiment were collected from surgical patients at Renmin Hospital of Wuhan University from June 2019 to June 2022. All tissues were placed in liquid nitrogen or 4% paraformaldehyde after isolation. The Ethics Committee of Renmin Hospital of Wuhan University approved this study. Procell provided the PCa cell line (DU145, PC3) required for our experiments (PLST, Wuhan, China). DU 145 cells were grown at 37 °C in RPMI-1640 (HyClone, Logan, UT, USA) culture medium containing 10% (volume/volume) fetal bovine serum (FBS) and was supplemented with 1% antibiotics (penicillin and streptomycin). In contrast, PC 3 cells were grown in Ham’s F-12K (, HyClone, Logan, UT, USA) culture medium containing 10% FBS, and all were incubated under 5% CO_2_.

### 2.2. Western Blotting

Cells or tissue samples were treated on ice for 30 min with RIPA lysis solution. Protease inhibitors were added to the RIPA buffer, and finally, the concentration of sample proteins was measured using the BCA test. All protein samples were stored at −20 or −80 °C. Medium amounts of protein per sample were separated and electro-transferred onto PVDF membranes (Millipore, NJ, USA) using 12% SDS polyacrylamide gels at 80 V and a current of 200 mA. β-actin was used as an internal reference to exclude differences caused by the larger sample size. Table 1 lists the antibodies used in this investigation. The uncropped blots and molecular weight markers are shown in Supplementary Figure S1.

### 2.3. Immunohistochemistry

Antigen retrieval was accomplished by deparaffinizing, dehydrating, and boiling sections. After sealing the slices with a 3% hydrogen peroxide solution, the primary antibody FAM107A was added and incubated at four degrees centigrade for more than 24 h (1:200 dilution). Finally, DAB chromogen and hematoxylin were applied to the slices. Two experienced pathologists assessed the immunostaining results and were not informed of specimen information before assessment.

### 2.4. Quantitative Real-Time PCR 

Total RNA from cells or tissue was extracted using TRI Reagent (Absin, Shanghai, China). cDNA was synthesized by employing the PrimeScript™ RT Reagent Kit (TaKaRa, Beijing, China) in accordance with the manufacturer’s instructions. TB Green^®^ Premix Ex Taq™ II (TaKaRa, Beijing, China) was utilized for the qRT-PCR assay. The primer sequences in our study were as follows (Sangon Biotech, Shanghai, China): β-Actin forward, 5′-TGATGGTGGGAATGGGTCAG-3′; β-Actin reverse, 5′-GGTGTGGTGCCAGATCTTCT-3′. FAM107A forward, 5′-TGCGGAGAATTGCCACACTGAC-3′; FAM107A reverse, 5′-AGAAGGGCTGAAGGAGGCTGTC-3′.

### 2.5. Methylation-Specific PCR (MSP)

Methyl Primer Express software was used to build MSP primers to explore the methylation status of FAM107A in the promoter region of PCa samples (Table 2). A cellular genomic DNA extraction kit (Servicebio, Wuhan, China) was used to extract genomic DNA from DU 145 and PC 3 cells, which were later treated and purified using bisulfite, followed by amplification and electrophoresis. We also used the 5′-aza deoxycytidine (5-Aza) reagent to treat DU 145 and PC 3 cells.

### 2.6. Cell Transfection 

The FAM107A overexpression lentivirus was constructed by OBio Biotechnology (Shanghai, China). The company also provided the control lentivirus plasmid (vector). PCa cells were grown overnight in six-well plates and infected with lentiviruses containing polybrene (8 g/mL). The original medium was replaced with medium containing 3 μg/mL puromycin after 24 h of cell infection, the uninfected PCa cells were removed, and the successfully infected cell lines were chosen for further research. Small interfering RNA targeting DNMT1 (RiboBio, Guangzhou, China) was synthesized and introduced into cells to generate si-DNMT1 cells. Western blotting was used to assess transfection efficiency. Specific information on small interference RNA is shown in Table S1.

### 2.7. Cell Proliferation Assay Cells 

The cells were first seeded in 96-well plates at a density of 2000 cells per well. Each well was filled with media containing 10 μL of CCK-8 (Boster, Pleasanton, CA, USA). The absorbance of each well was measured at 450 nm after 2 h of incubation at 37 °C to determine cell proliferation. Furthermore, cell proliferation was detected using the Ki67 immunofluorescence assay.

### 2.8. Colony Assay

In six-well plates (Servicebio, Wuhan, China), cells were uniformly inoculated, and the cell density in each well was kept at 600 cells/μL. After 14 days of cell culture, the cells were fixed with 4% paraformaldehyde, stained with gentian violet, and washed with phosphate-buffered saline. Visible colonies were counted, and the colony formation ability was compared.

### 2.9. Transwell Invasion Analysis

In serum-free medium, DU145 cells or PC3 cells were seeded in the upper chamber at the top end of a Transwell plate (8 μm pore size) containing Matrigel. The number of DU 145 cells per well was kept at 3 × 10^4^, while the number of PC 3 cells was kept at 5 × 10^4^. A total of 750 µL of culture medium containing 10% FBS was introduced into the bottom compartment.

Cells were fixed in paraformaldehyde and stained with gentian violet after two days of cell culture. Next, the Transwell chambers were dried in an oven. Finally, a microscope was used to count the number of cells that migrated to the subsurface membrane.

### 2.10. Wound Healing Assay

The cells were first cultured in a six-well plate until they reached 85% cell density. Then, the six-well plate was scratched vertically using a 200 µL pipette tip to make a wound. After three washes in PBS, the culture was continued with 2% FBS-containing culture medium. At the beginning and after 24 h, images of the wound in a six-well plate were captured under a microscope.

### 2.11. Immunofluorescence

The appropriate density of DU 145 and PC 3 cells was inoculated on slides, fixed with paraformaldehyde, blocked with goat serum, and incubated with primary antibody for more than 24 h at 4 °C. The next day, the cells were incubated with a fluorescently labeled secondary antibody for an additional 70 min, and finally, nuclear staining was performed with DAPI.

### 2.12. Flow Cytometry 

When the cell growth density of each group reached 80%, PBS-washed cells were collected in centrifuge tubes and fixed in 70% ethanol overnight. Cells were again washed with PBS the following day, and cell cycle changes were detected using PI flow cytometry staining after fluorescent labeling of the cells. 

When the cell growth density of each group reached 85%, PBS was used to wash the cell supernatant, and the pellets were collected in centrifuge tubes; after fluorescence labeling of the cells, whether the cell membrane was intact was detected by binding of phospholipid-binding protein to phosphatidylserine in a flow cytometer to determine apoptosis.

### 2.13. Xenograft Assay

Ten 4-week-old, sex-identical BALB/c mice were purchased from Hunan Slike Jingda Laboratory Animals (Changsha, China) to construct the in vivo model. BALB/c nude mice were randomly separated into the pc-Vector and pc-FAM107A groups and housed in a particular pathogen-free environment (SPF) at the Renmin Hospital of Wuhan University’s animal experimental base. One week before the start of the experiment, BALB/c mice were acclimated to the SPF. Then, DU 145 cells transfected with the PC vector and pcFAM107A were injected into the axils of BALB/c mice. These DU 145 cells were diluted in 150 L of serum-free medium with a cell number of approximately 10^6^. They were measured and recorded every 5days and then euthanized 1 month later. After dissection and photographic recording, tumor tissues were stored in liquid nitrogen or formaldehyde.

### 2.14. Statistical Analysis

SPSS 22.0 and GraphPad Prism 8 were used to perform the statistical analysis. Student’s *t* test or chi-square tests were used to analyze the data. The Kaplan–Meier method was used to construct survival curves that were then statistically examined using the logarithmic-rank test. Unless otherwise stated, the results are provided as the mean ± SD. Statistical significance was defined as a *p* value of <0.05. *** for *p* < 0.001, ** for *p* < 0.01, and * for *p* < 0.05.

## 3. Result

### 3.1. FAM107A Expression and Prognostic Analysis in Prostate Tissue

**Figure 1:**
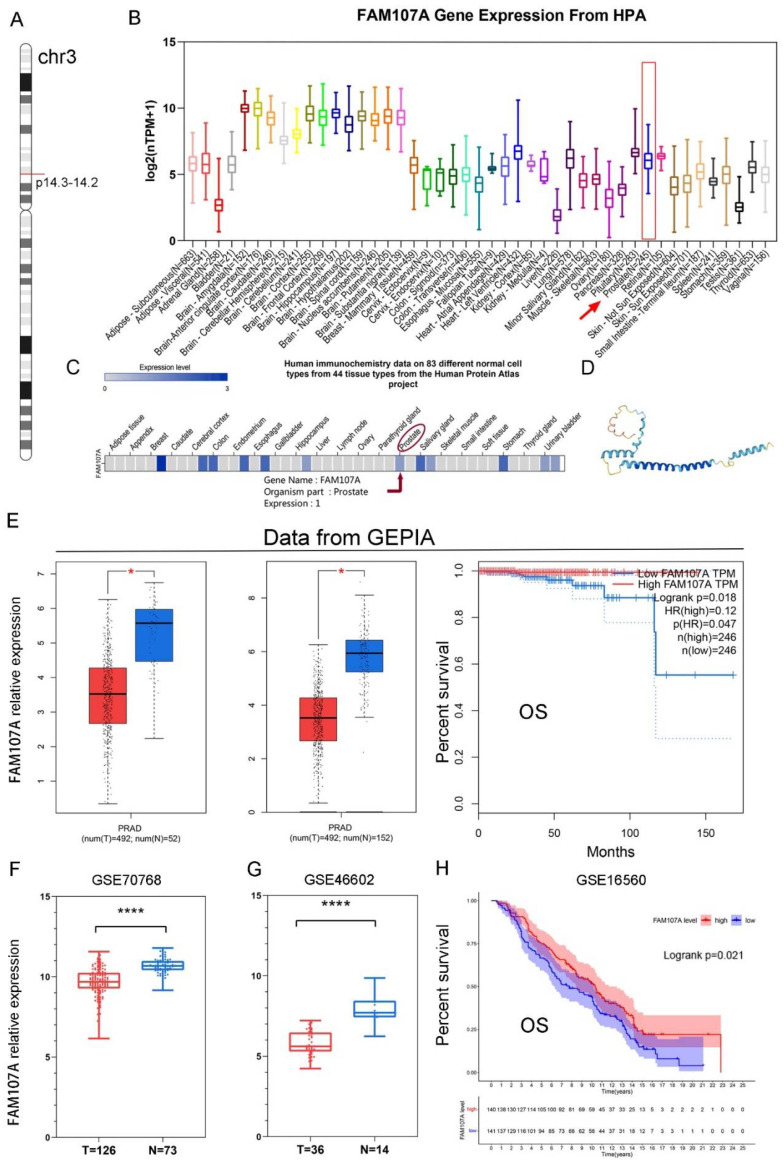
Chromosomal localization of FAM107A in PCa, expression characteristics of FAM107A gene, and its correlation with clinical prognosis. (**A**) FAM107A is located in the short-arm region of chromosome 3. (**B**) Tissue expression of human FAM107A gene. Distribution of RNA expression of FAM107A gene in normal tissue types. It was created using the transcriptomic dataset of the Human Protein Atlas database. (**C**) FAM107A protein expression in prostate tissue according to human immunochemistry data on 44 tissues from the Human Protein Atlas project. (**D**) Prediction of the possible 3D structure of the FAM107A protein according to AlphaFold Monomer v2.0. (**E**) FAM107A was expressed at lower levels in prostate tumor specimens than normal prostate tissue specimens. GEPIA includes 492 prostate cancer tissue samples and 52 normal prostate tissue samples from the TCGA database, and 100 normal prostate tissue samples from the GTEx database. Overall survival (OS) of patients with prostate cancer according to FAM107A expression (http://gepia.cancer-pku.cn (accessed on 1 June 2022)). (**F**) FAM107A expression levels were lower in prostate cancer tumor tissues compared to normal prostate tissues in the GSE70768 dataset. (**G**) FAM107A expression levels were lower in prostate cancer tumor tissues compared to normal prostate tissues in the dataset GSE46602. (**H**) Kaplan–Meier analysis of overall survival (OS) of prostate cancer patients according to FAM107A expression in the GSE16560 dataset. **** for *p* < 0.0001, and * for *p* < 0.05.

As shown in Figure 1A, the FAM107A gene is located on chromosome 3 p14.3-p14.2. Figure 1B shows the expression of the FAM107A transcript. Figure 1C shows the protein levels of FAM107A, while Figure 1D shows the predicted 3D protein structure. In addition to higher levels of expression levels in brain tissue, FAM107A is moderately expressed in normal prostate tissue.

The expression of FAM107A was much lower in PCa tissues, according to the GEPIA website. Differential analysis using TCGA alone or in combination with the GTEx database revealed that FAM107A expression was lower in PCa than in normal tissues. FAM107A expression was inversely associated with overall patient survival (OS) (*p* = 0.018) (Figure 1E). FAM107A expression in PCa tissues was significantly lower than that in normal tissues, according to GSE70768 and GSE46602 datasets (Figure 1F,G). Another dataset confirmed the survival analysis results (GSE16560), where low expression of FAM107A in PCa was associated with poorer OS (*p* = 0.021) (Figure 1H).

**Figure 2:**
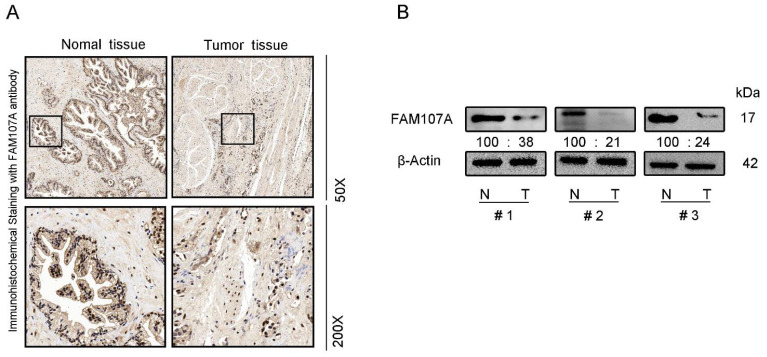
FAM107A expression was higher in normal prostate tissues than in prostate cancer tissues. (**A**) Measurement of protein levels of FAM107A in normal and prostate cancer tissues by immunohistochemical analysis. Representative images were shown at magnifications of 50× and 200×, respectively. (**B**) Protein levels of FAM107A in normal and prostate cancer tissues by Western blotting.

We also performed immunocytochemical staining for FAM107A in prostate tissues. FAM107A showed strong positive expression in normal prostate tissues, while positive expression was lower in PCa tissues (Figure 2A). Furthermore, the expression of FAM107A in PCa tissues was detected by Western blotting, and the results indicate that the expression of FAM107A in tumor tissues was lower than that in normal prostate tissue (Figure 2B).

### 3.2. DNA Promoter Methylation Was Responsible for the Downregulation of FAM107A Expression

**Figure 3:**
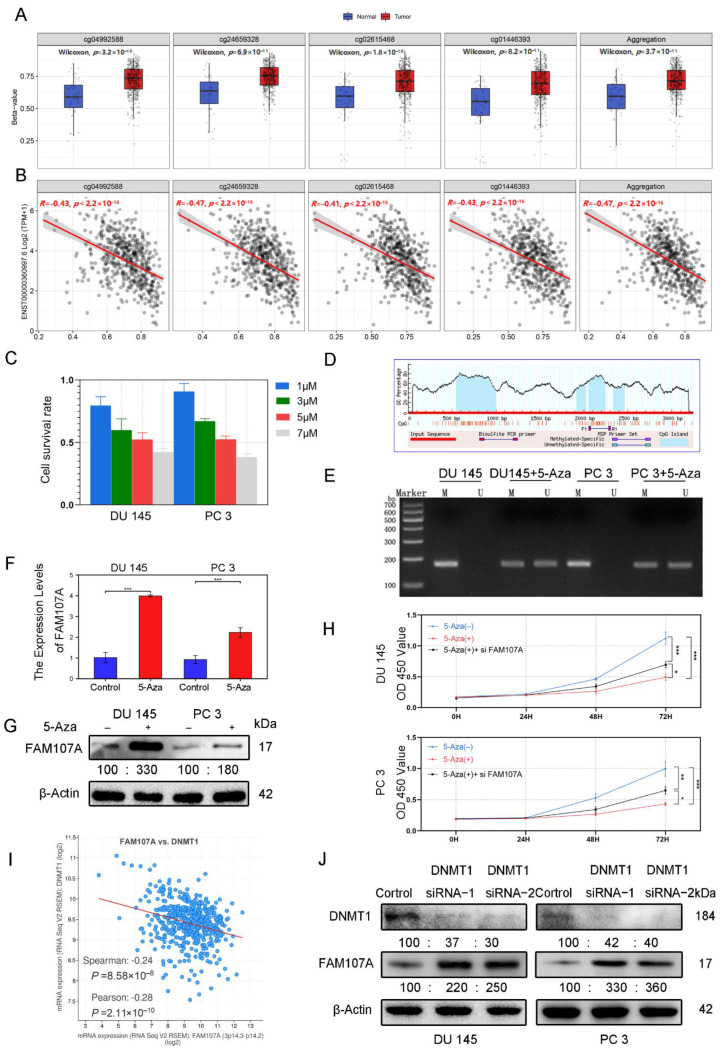
FAM107A exhibits low expression in prostate cancer cells due to hypermethylation of its promoter region. (**A**) cg04992588, cg24659328, cg2615468, and cg01446993 were used as probes to detect the promoter methylation level of the FAM107A gene in normal and tumor tissues (http://www.bioinfo-zs.com/smartapp/ (accessed on 1 June 2022)). (**B**) cg04992588, cg24659328, cg2615468, and cg01446993 probes were negatively correlated with FAM107A gene expression. (**C**) Growth inhibitory effect of different concentrations of 5-Aza on prostate cancer cells. (**D**) The methylation region of the promoter (CpG island) of the primer. (**E**) MSP analysis of the methylation status of the FAM107A promoter before and after 5-azacytidine treatment. “ U “indicates amplification without methylation, and” M “indicates amplification with methylation. (**F**) 5-Aza treatment rescues the mRNA expression of FAM107A. (**G**) Effect of 5-azacytidine on FAM107A protein expression in prostate cancer cells by Western blotting. (**H**)Analysis of the effects of 5-Aza and FAM107A siRNA on cell proliferation according to a CCK8 assay. (**I**) Correlation between FAM107A and DNMT1 was analyzed using the prostate cancer dataset included in the Cbioportal database (https://www.cbioportal.org/ (accessed on 1 June 2022)). (**J**) Effect of DNMT1 on FAM107A protein expression in prostate cancer cells as determined by Western blotting. *** for *p* < 0.001, ** for *p* < 0.01 and * for *p* < 0.05.

According to the analysis of the Smartapp database, multiple probes in the FAM107A promoter region showed higher β values in PCa tissues than in normal tissues and a decreasing trend of FAM107A expression with increasing β values (Figure 3A). We suspect that this hypermethylation of the promoter region causes a reduction in FAM107A expression (Figure 3B). Different concentrations of 5-Aza inhibited the growth of prostate cancer cells after 48 h of treatment, and the proliferation ability of prostate cancer cells decreased with increasing drug concentration. We chose an appropriate concentration (5 μM) for the next step of exploration (Figure 3C). We used Methyl Primer Express software to construct primers for the methylation region of the promoter (CpG island) (Figure 3D). We tested the above hypothesis in PCa cells DU145 and PC3 using the 5-Aza methyltransferase inhibitor 5-aza. According to the MSP results, amplified fragments could be obtained from primers for nonmethylation of 5-Aza-treated cells, indicating a decrease in methylation at this locus. Exposure to 5-Aza reduced the levels of methylation of the FAM107A promoter region in PCa cells (Figure 3E). According to the PCR and the Western blotting results, the expression of FAM107 in DU145 and PC3 cells was partially restored after 5-Aza treatment (Figure 3F,G). The effects of 5-Aza and FAM107A siRNA on cell proliferation ability were further verified. The results show that the inhibitory effect of 5-Aza on cell proliferation decreased after interfering with FAM107A expression (Figure 3H).

Given the importance of DNA methyltransferase 1 (DNMT1) in methylation, the Cbioportal database tentatively confirmed a negative correlation between FAM107A and DNMT1 expression. (Figure 3I). After knockdown of DNMT1 with siRNA, DNMT1 expression was downregulated in DU145 and PC3 cells, while AM107A expression was partially restored (Figure 3J). In conclusion, these results suggest that hypermethylation of the FAM107A promoter region inhibited its expression in prostate cancer cells and that DNMT1 was involved in maintaining hypermethylation.

### 3.3. Overexpression of FAM107A Inhibited PCa Cell Growth and Invasion In Vitro

**Figure 4:**
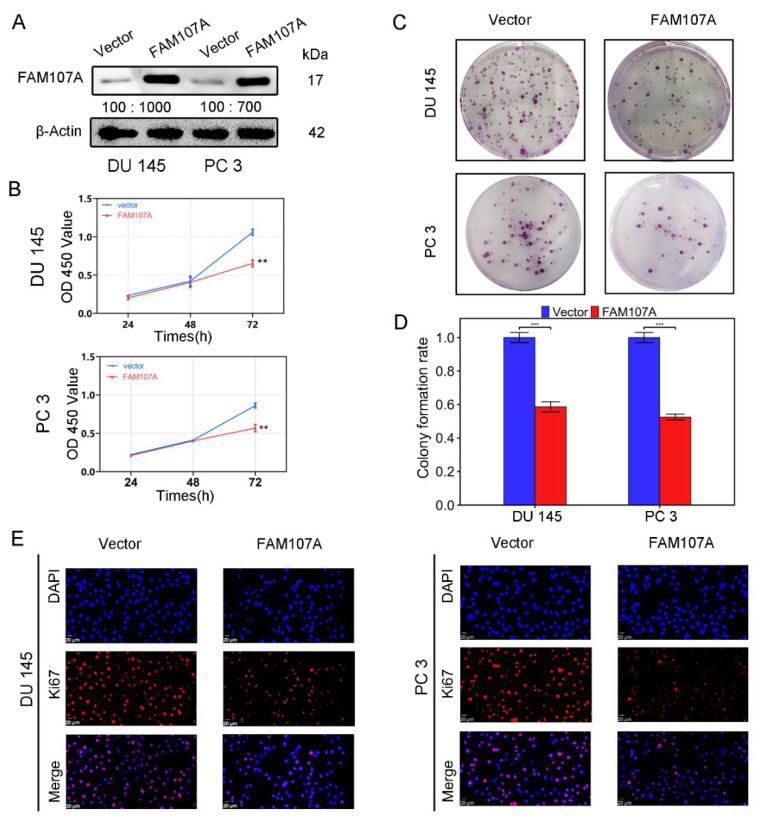
FAM107A overexpression inhibited the proliferation of PCa cells in vitro. (**A**) The Western blotting analysis confirmed the effect of FAM107A overexpression. The unit of molecular weight is kDa. (**B**) Comparison of the proliferative capacity of the two groups of cells, detected using the CCK-8 kit, with OD values measured every 24 h. (**C**,**D**) Comparison of the colony-forming ability of the two groups of cells. Colony formation was quantified 14 days after colony formation using ImageJ software. (**E**) Immunofluorescence was used to detect Ki67 expression in the cells at a magnification of 400×. Similar results were obtained, and data are expressed as the mean ± SD of at least three experiments. *** for *p* < 0.001, and ** for *p* < 0.01.

Using an in vitro study, we analyzed the effects of FAM107A overexpression on the biological behavior of PCa cells DU145 and PC3. Cells stably expressing the FAM107A gene were successfully created. According to the Western blotting results, the expression level of FAM107A in the pc-FAM107A group was significantly higher than that in the pc-vector group (Figure 4A).

The CCK-8 assay showed that the cell proliferation ability of the pc-FAM107A group was significantly decreased compared to that of the pc-Vector group starting from the third day, indicating that the overexpression of FAM107A inhibited the proliferation and growth of prostate cancer cells (*p* < 0.01) (Figure 4B). A 14-day colony formation assay also showed that FAM107A overexpression reduced the colony formation ability of PC3 and DU145 cell lines (*p* < 0.001) (Figure 4C,D). The results of the Ki67 immunofluorescence assay also confirm that the Ki67 expression of cells in the pc-FAM107A group was lower than that of cells in the pc-Vector group (Figure 4E).

**Figure 5:**
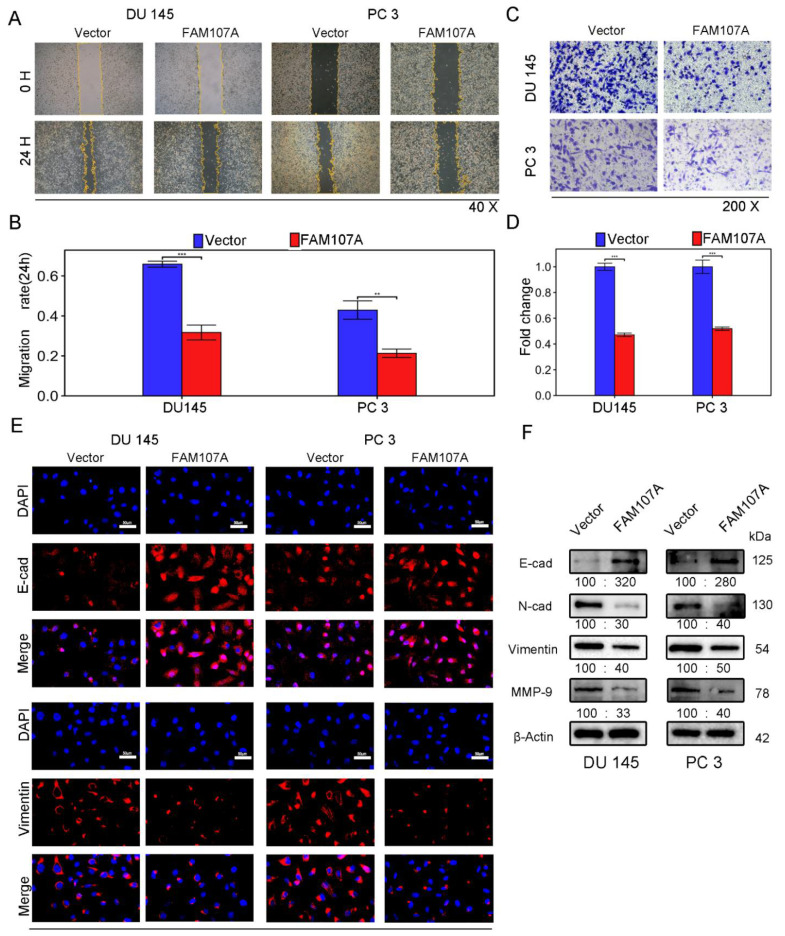
Overexpression of FAM107A inhibited PCa cell migration in vitro as well as invasion. (**A**,**B**) The migration ability of cells was assessed using a wound healing assay. The healing area of wounds was quantified by ImageJ software 24 h after wound formation. (**C**,**D**) Cell invasiveness was assessed using a Transwell invasion assay. The number of invaded PCa cells was quantified with ImageJ software. (**E**) Inhibition of EMT by overexpressed FAM107 was verified by Western blotting, and molecular weights are in kDa. (**F**) Immunofluorescence assays were performed to detect E-cad and Vimentin expression in cells at a magnification of 400×. Data are expressed as the mean ± SD of at least three experiments. *** for *p* < 0.001, and ** for *p* < 0.01.

The wound healing assay revealed that 24 h after FAM107A overexpression, the area of wound healing was considerably decreased in DU145 and PC3 cells, and the difference was statistically significant (Figure 5A,B). FAM107A overexpression also decreased the number of migrating cells, according to Transwell invasion studies. This showed that the cell capacity to invade was hampered (Figure 5C,D).

Next, we investigated whether there was a link between FAM107A and epithelial–mesenchymal transition (EMT). We observed the expression of traditional epithelial markers (E-cadherin) and mesenchymal markers (N-cadherin, vimentin, matrix metalloproteinase-9) markers before and after FMA107A overexpression by immunofluorescence and Western blotting. Overexpression of FAM107A resulted in increased intracellular E-cadherin (E-cad) expression and decreased N-cadherin (N-cad), matrix metalloproteinase-9 (MMP-9), and vimentin expression, according to the findings. (Figure 5E,F).

Overall, overexpression of FAM107A decreased PCa proliferation, migration, and invasion.

### 3.4. Regulation of the Cell Cycle and Apoptosis by FAM107A

**Figure 6:**
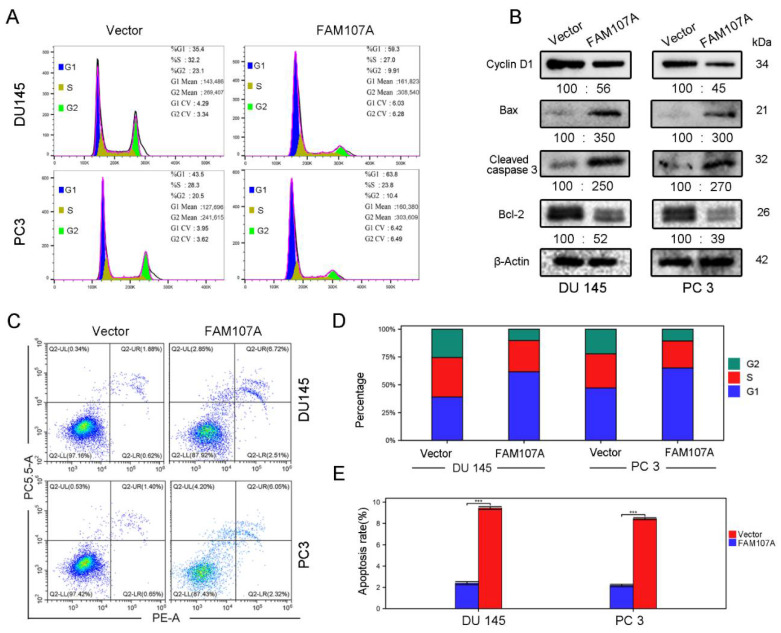
Overexpression of FAM107A promotes prostate cancer cell cycle arrest and induces apoptosis in prostate cancer cells. (**A**) Detection of the effect of FAM107A on the cycle of DU 145 and PC 3 cells using flow cytometry. (**B**) Western blotting to verify changes in cyclin D1 and apoptosis markers Bax, Cleaved caspase-3, and Bcl-2. (**C**) Detection of apoptosis rate of both groups of cells using flow cytometry. (**D**,**E**) Quantitative analysis of cell cycle changes and changes in apoptosis rates. Data are expressed as the mean ± standard deviation of at least three experiments. *** for *p* < 0.001.

Flow cytometry was used to detect changes in the cell cycle and the apoptosis rate of DU145 and PC3 cells after FAM107A overexpression. In the PC-FAM107A group, the fraction of cells in phase G1 increased dramatically in the DU145 and PC3 cell lines (Figure 6A,D), showing that cells were blocked in phase G1 after FAM107A overexpression. The results of flow cytometry further reveal that the proportion of apoptotic cells in the PC-FAM107A group was higher than in the PC-vector group (Figure 6C,E). Overexpression of FAM107A lowered the production of G1-phase-associated protein (Cyclin D1), increased the production of apoptotic indicators Bax and cleaved caspase-3, and decreased the expression of Bcl-2, according to Western blotting data (Figure 6B).

### 3.5. In PCa Cells, FAM107A Suppressed the FAK/PI3K/AKT Signaling Pathway by Downregulating FAK Expression

**Figure 7:**
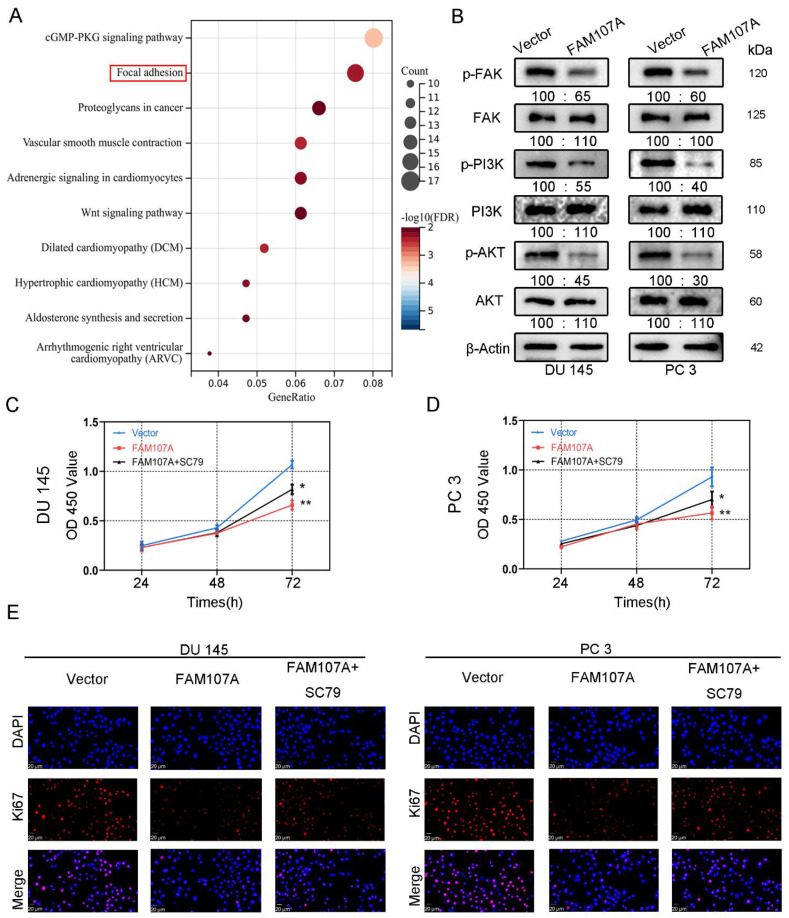
FAM107A affects PCa cell proliferation through FAK/PI3K/AKT pathway. (**A**) Enrichment results of the KEGG pathway suggest that FAM107A may be involved in PCa progression through the FAK/PI3K/AKT pathway. (**B**) Western blotting analysis of p-FAK expression and expression of downstream signaling pathway proteins p-PI3K and p-AKT. (**C**) AKT activator SC79 could partially restore the inhibition of cell proliferation ability after FAM107A overexpression. (**D**) The CCK-8 experiment was used to compare the proliferation activity of PC3 and DU145 cells in different groups. (**E**) An immunofluorescence assay was performed to detect the expression of Ki67 in each group of cells at a magnification of 400×. ** for *p* < 0.01, and * for *p* < 0.05.

To demonstrate how FAM107A regulates the progression of PCa, we first performed an enrichment analysis of the KEGG pathway using the CBioPortal database (Prostate Adenocarcinoma-Firehose Legacy). The results show that FAM107A could regulate prostate cancer progression through the focal adhesion kinase (FAK) pathway (Figure 7A). The results of Western blotting verify the above speculation. In addition to the reduced expression of p-FAK, the downstream pathways of FAK, such as phosphorylated PI3K and phosphorylated Akt, were also similarly reduced. No significant changes were observed in T-FAK, T-PI3K, or T-Akt protein levels (Figure 7B).

We next activated AKT to observe prostate cancer cell proliferation and invasive capability. FAM107A overexpression was attenuated after AKT activation, and the proliferation and invasive capacity of the cells were enhanced. Treatment of cells with 20 μM SC79, an activator of Akt phosphorylation, for 24 h partially counteracted the inhibitory effect of FAM107A on PCa cell proliferation, migration, and invasion. In the CCK8 assay, SC79 was able to reverse suppression of cell proliferation by FAM107A (Figure 7C,D). The results of the Ki67 immunofluorescence experiments are also consistent with this finding (Figure 7E).

**Figure 8:**
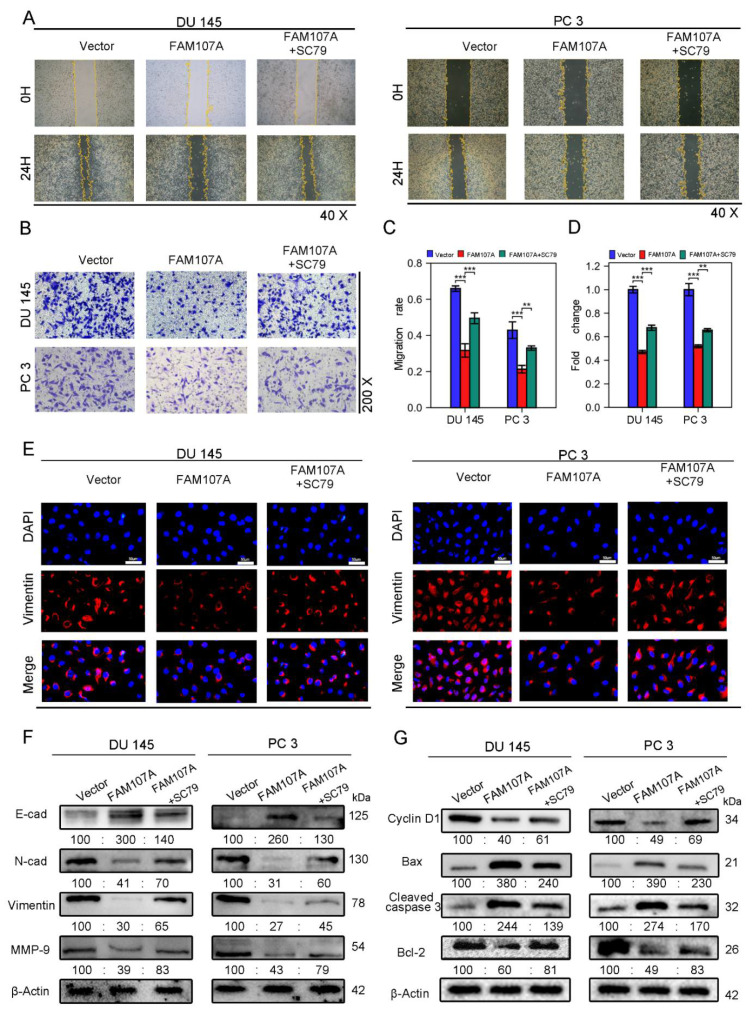
FAM107A affects PCa cell migration and invasion through FAK/PI3K/AKT pathway. (**A**,**B**) AKT activator SC79 partially restores the effect of FAM107A overexpression on cell invasion ability. The number of invading PCa cells was quantified using ImageJ software. (**C**,**D**) Effect of AKT activator SC79 partially restores FAM107A overexpression on wound healing assays. Wound healing was quantified using ImageJ software. (**E**) An immunofluorescence assay was used to detect the expression of Vimentin in each group of cells at a magnification of 400×. (**F**) Western blotting analysis of changes in key proteins during EMT. (**G**) Western blotting analysis of changes in key proteins during cell cycle and apoptosis.*** for *p* < 0.001, and ** for *p* < 0.01.

SC79 also increased the area of scratch wound healing in the wound healing assay (Figure 8A,C) and the number of invading cells in the Transwell invasion test (Figure 8B,D). Because phosphorylated AKT induces the expression of EMT markers, and we have previously demonstrated that overexpression of FAM107A results in increased expression of E-cadherin and decreased expression of N-cadherin, Vimentin, and MMP-9, we suspected that SC79 could antagonize the effects of FAM107A overexpression. The Western blotting results confirm this conjecture. Under the action of SC79, we detected a partial decrease in E-cadherin expression and a partial increase in the expression of N-cadherin, Vimentin, and MMP-9. And Vimentin immunofluorescence experiments confirmed the above changes (Figure 8E,F).We further explored the changes in the cell cycle and apoptosis-related proteins following the use of SC79. SC79 could partially antagonize cyclin D1, as well as changes in Bax, cleaved caspase-3, and Bcl-2 expression(Figure 8G). 

All of the above findings suggest that FAM107A can inhibit PCa progression via the FAK/PI3K/AKT pathway.

### 3.6. The Inhibitory Effect of FAM107A on PCa Cells In Vivo

**Figure 9:**
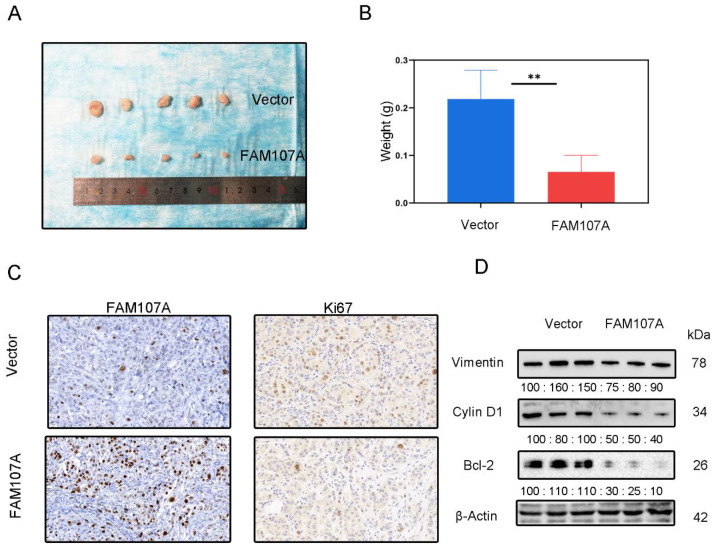
Overexpression of FAM107A inhibited the growth of PCa cells in vivo. (**A**) Comparison of tumor model sizes in the pc-Vector pc-FAM107A group. (**B**) Comparison of the weight of tumors in the pc-Vector pc-FAM107A group. (**C**) Ki67 and FAM107A expression in tumor tissues was detected by immunohistochemistry. (**D**) Western blotting was used to analyze the changes in Vimentin, cyclin D1, and Bcl-2 proteins in the tumors of the pc-FAM107A pc-Vector group. ** for *p* < 0.01.

DU145 cells expressing pc-Vector or pc-FAM107A were injected into mice to determine whether FAM107A can decrease cancer growth in vivo. We discovered that mouse tumor models injected with pc-FAM107A cells were much smaller than those in the control group thirty days after the start of the experiment (Figure 9A). The average tumor weight of tumor cells in mice injected with pc-FAM107A cells was only half that of the control group (Figure 9B). The immunohistochemical results also show a significant decrease in Ki67 expression in tumors of the pc-FAM107A-injected group (Figure 9C,D). We also used Western blotting to confirm that Vimentin, cyclin D1, and Bcl-2 were reduced in the pc-FAM107A group (Figure 9D).

**Figure 10:**
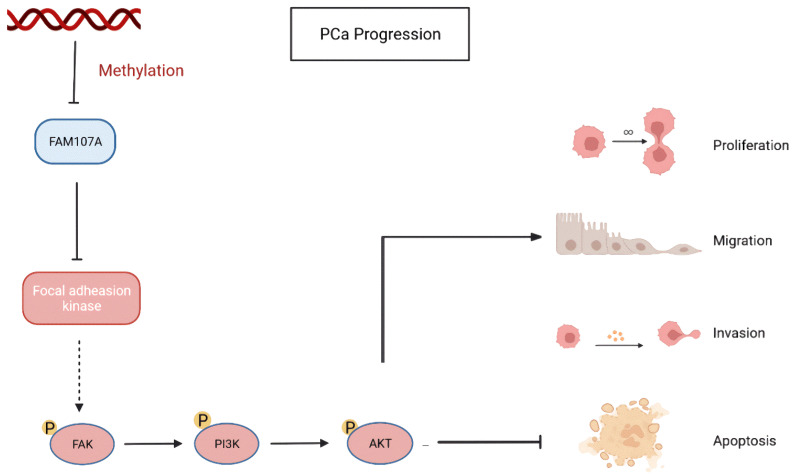
Inactivation of FAM107A associated with promoter methylation promotes prostate cancer progression through the FAK/PI3K/AKT pathway.

Finally, using a xenograft mouse model, we showed that FAM107A efficiently suppressed the development of PCa tumors in vivo. Based on the above results, FAM107A inactivation associated with promoter methylation affects prostate cancer progression through the FAK/PI3K/AKT pathway (Figure 10).

## 4. Discussion

The position of FAM107A on chromosome 3p14.3-p14.2 is characteristic, as several reports have indicated that genes in the short-arm area of chromosome 3, for the most part, have been identified and confirmed as TSGs. These genes are normally expressed in normal tissues but are inactivated in cancerous tissues [14,15,16]. The expression of FAM107A in normal tissues matched the above characteristics. Of these, FAM107A expression was modest in noncancerous prostate tissues and markedly downregulated in PCa tissues.

TSG activation can be induced by various factors, including epigenetic modifications (DNA methylation, histone modifications) and mutations (chromosomal mutations, point mutations), which play indispensable roles in its activation [17,18]. DNA methylation refers to the addition of a methyl group to the cytosine 5′-carbon atom by the action of DNA methyltransferase, resulting in the formation of 5-methylated cytosine. TSG transcriptional silencing typically occurs when DNA methylation occurs in the CpG island of gene promoter [19]. DNA methyltransferase (DNMT) family members include DNMT1, DNMT3A, and DNMT3B. DNMT1 is mainly responsible for maintaining DNA methylation, and various studies have shown that DNMT1 depletion leads to extensive demethylation of gene promoter regions [20,21]. DNA promoter hypermethylation can also be reversed by demethylating drugs, which can also provide new agents for cancer treatment [22]. According to the data analysis on the SMART website, the degree of methylation in the promoter region of FAM107A increases in PCa, and the expression of FAM107A decreases with increasing degrees of methylation.

In this study, the expression of FAM107A in DU 145 and PC 3 cell lines was restored to some extent by treating prostate cancer cells with the demethylating drug 5-Aza [23] or by knocking down the expression of DNMT1 in siDNMT1 cells. This finding is in agreement with a previous report by Donkena et al. [24]. Additionally, it also supports the hypothesis that DNA methylation is at least one of the critical molecular processes suppressing FAM107A expression in PCa cells.

TSGs normally encode proteins that are responsible for delaying malignant transformation and oncogenic advancement. They can negatively regulate cell cycle progression, inhibit metastatic dissemination of tumor cells, and induce apoptosis [25]. This gene is also called DRR1 because when a fragment of it was introduced into renal cell carcinoma cells, it inhibited tumor development and invasion. It was recently discovered to play a cancer-suppressor role in bladder cancer [10,11]. However, few detailed studies are available in the literature on the mechanism of action of FAM107A, with only the simple conclusion that FAM107A acts as a TSG. However, another study showed that FAM107A could promote glioblastoma progression and enhance the migration capacity of glioblastoma cells [26]. It is surprising that this investigation suggested that FAM107A may differ in regulating the biological functions of different tumors. The GEPIA database revealed that FAM107A expression was reduced in PCa tissues, and survival analysis revealed that high levels of FAM107A expression were associated with a favorable OS rate in patients with prostate cancer. We also validated the above results using three independent datasets, GSE70768, GSE46602, and GSE16560, from the GEO database [27,28,29]. These findings are similar to those of a previous study by Ma et al., all indicating that FAM107A expression is downregulated in PCa or is linked to a worse prognosis [30]. However, what exactly is the specific mechanism of FAM107A activity in prostate cancer? This was the second objective of this study.

We revealed that restoring FAM107A expression in prostate cancer cell lines such as DU 145 and PC 3 inhibited cell cycle progression, increased the proportion of cells in G1, and inhibited the expression of cyclin D1, a key protein in the G1 phase. It also inhibited cell proliferation, migration, and invasion and resulted in apoptosis. EMT, defined as the process by which tumor cells acquire properties of mesenchymal cells, is a critical step for cancer cell recurrence and metastasis and is associated with tumor cell migration in vitro [31,32]. Downregulation of E-cadherin and upregulation of mesenchymal markers, including N-cadherin and wave proteins, are molecular markers of EMT. Additionally, the matrix metalloproteinase family (MMP) is involved in the EMT process in different types of cancer [33]. According to our findings, the overexpression of FAM107A elevated E-cadherin expression, reduced N-cadherin and vimentin expression, and interfered with EMT in tumor cells. The expression of MMP-9 was also found to decrease after FAM107A overexpression, suggesting that FAM107A overexpression reduces tumor aggressiveness. These results indicate that FAM107A might influence the EMT process in PCa cells.

This study also found that FAM107A inhibits PCa cell proliferation, migration, and invasion by acting on the FAK/PI3K/AKT pathway. Furthermore, we used KEGG enrichment analysis to explore how FAM107A influenced PCa progression, and the results suggest that FAM107A is related to FAK phosphorylation, which is abnormal. As a membrane receptor, FAK is an early step in numerous intracellular signaling pathways. PI3K/AKT is an important downstream pathway of FAK that is activated by FAK and therefore affects the biological process of tumor cells [34,35,36]. Activation of the PI3K/AKT pathway has been implicated in a variety of biological activities in various tumors, including PCa, cell proliferation, migration, and invasion. Activation of PI3K/AKT and its downstream mTOR/p70 signaling pathway can regulate the expression of cell-cycle-related proteins, accelerate cell cycle progression in the G1 phase, and affect PCa cell proliferation [37]. In PCa cells, the PI3K/AKT and downstream mTOR/S6K1 signaling pathways are inhibited, resulting in cell apoptosis [38]. AKT will also undergo abnormal phosphorylation under the action of PGE2 and TGF-β, leading to changes in PI3K/AKT and downstream mTOR pathways and accelerating the EMT process of cells [39]. Taken together, these findings indicate that although the mechanisms of PI3K/AKT during PCa cell proliferation, apoptosis, migration, and invasion are not identical, PI3K/AKT is a key link in key signaling pathways in PCa.

Overexpression of FAM107A significantly inhibited the expression levels of phosphorylated FAK but lowered the expression of p-PI3K and p-Akt. FAM107A may influence tumor progression by blocking the FAK/PI3K/AKT signaling cascade. We performed subsequent complementary experiments and found that the Akt activator SC79 reversed the inhibitory effect of FAM107A on PCa cell proliferation and migration. Finally, it was confirmed that FAM107A affects PCa initiation and growth by inhibiting the FAK/PI3K/AKT signaling pathway.

## 5. Conclusions

Our findings suggest that FAM107A can function as a TSG in PCa; its inactivation is caused by promoter hypermethylation. FAM107A affects the development and progression of PCa via the FAK/PI3K/AKT signaling pathway. Therefore, FAM107A is important in at least two aspects of the therapeutic outlook of PCa. Overexpression of FAM107A has the potential to be used as a method of treatment for PCa, and therapies that target epigenetic events that regulate the expression of FAM107A could also potentially be an effective strategy for treating PCa. However, further exploration is also needed to provide more in-depth findings on its specific roles in cancer development and to expand its clinical application.

## Figures and Tables

**Table 1:** Primary antibodies used in this research.

Antibody	Specificity	WB	IF	IHC	Company
FAM107A	Rabbit	1:500	-	1:200	Proteintech
DNMT1	Rabbit	1:500	-	-	Proteintech
E-cadherin	Rabbit	1:1000	1:100	-	Proteintech
N-cadherin	Rabbit	1:1000	-	-	Proteintech
Vimentin	Rabbit	1:1000	1:100	-	Proteintech
MMP-9	Rabbit	1:1000	-	-	Proteintech
FAK	Rabbit	1:1000	-	-	ABclonal
p-FAK	Rabbit	1:1000	-	-	ABclonal
PI3K	Rabbit	1:1000	-	-	ABclonal
p-PI3K	Rabbit	1:1000	-	-	ABclonal
AKT	Mouse	1:1000	-	-	ABclonal
p-AKT	Mouse	1:1000	-	-	ABclonal
Cyclin D1	Rabbit	1:1000	-	-	Proteintech
Bax	Rabbit	1:1000	-	-	CST
Bcl-2	Rabbit	1:1000	-	-	CST
Cleaved caspase-3	Rabbit	1:1000	-	-	CST
Ki67	Rabbit	-	1:100	1:200	Abcam
β-actin	Rabbit	1:1000	-	-	Proteintech

**Table 2:** Primers for MSP analysis.

Primer Name	Sequence (5′-3′)	Length	Tm	GC Content (%)	Number of Bases
H-FAM107A (M)-F	TTTGGGATTTGGGGTCGC	181	61.2	55.6	18
H-FAM107A (M)-R	AACACAACCCGATAAAACCCG		61.5	47.6	21
H-FAM107A (U)-F	GGTTTGGGATTTGGGGTTGT		60.9	50	20
H-FAM107A (U)-R	ACAACACAACCCAATAAAACCCA	185	61.1	39.1	23

## Data Availability

The data presented in this study are availability in Supplementary Material here.

## Supplementary Materials

The following supporting information can be downloaded at: https://www.mdpi.com/article/10.3390/cancers14163915/s1, Table S1: sequences of the siRNAs.
