# Supplementary material for: FAM107A Inactivation Associated with Promoter Methylation Affects Prostate Cancer Progression through the FAK/PI3K/AKT Pathway
Source: Cancers (Basel). 2022 Aug 13;14(16):3915. doi: 10.3390/cancers14163915 (PMC9405870; doi:10.3390/cancers14163915)
Supplement: Supplementary file 1 [file cancers-14-03915-s001.zip › Table S1.pdf]

Supplementary Table S1. sequences of the siRNAs.

siRNAs	Sense Strand (5'-3')	
DNMT1 siRN-1	GGAAGAAGAGUUACUAUAAGA	UUAUAGUAACUCUUCUUCCCA
DNMT1 siRNA-2	GCUUCAGUGUGUACUGUAAGC	UUACAGUACACACUGAAGCAG
